# Nurses’ knowledge, attitudes, practices, and educational needs regarding frailty: a multicenter cross-sectional survey in southwestern China

**DOI:** 10.3389/fpubh.2026.1920309

**Published:** 2026-09-17

**Authors:** Jie Lyu, Jun Yang, Ling-Na Kong, Fen Xie, Ying Huang, Ping Liao

**Affiliations:** 1 Department of General Practice, The First Affiliated Hospital of Chongqing Medical University, Chongqing, China; 2 School of Nursing, Chongqing Medical University, Chongqing, China; 3 Chonggang General Hospital, Chongqing, China

**Keywords:** attitudes, continuing education, cross-sectional study, educational needs, frailty, knowledge, nurses, practices

## Abstract

**Background:**

Population aging has made frailty a major public health concern. Frailty is potentially reversible when identified and managed early, and nurses are well positioned to deliver such care; however, nurses’ frailty-related competencies vary considerably, and evidence remains scarce in southwestern China, where the aging burden is disproportionately high. We therefore conducted a multicenter study to assess nurses’ knowledge, attitudes, and practices (KAP) regarding frailty, explore their associated factors, and clarify their educational needs.

**Methods:**

An anonymous online survey was administered to a convenience sample of nurses at 41 tertiary general hospitals in Chongqing Municipality and Sichuan Province, China, between January and March 2025. Frailty-management competency was measured with a validated 31-item KAP questionnaire (total score 31–127; higher indicates better competency), and educational needs with an 11-item questionnaire. Associations were assessed using linear mixed-effects models with a random intercept for hospital; multivariable models entered 11 prespecified covariates, and reporting followed STROBE.

**Results:**

Of 637 nurses recruited, 610 (95.76%) provided valid questionnaires. The mean total KAP score was 103.07 ± 12.65 (81.16% of maximum). Attitude scored highest (88.53%), followed by knowledge (81.38%) and practice (74.40%), with practice corresponding to the lowest percentage of its maximum. In fully adjusted mixed-effects models, more recent frailty education was independently associated with the total KAP score (overall *p* = 0.019; never-trained vs. trained within the past month, *β* = −6.820, 95% CI − 13.220 to −0.430, *p* = 0.036), whereas the association with recency of frailty-related reading was borderline (overall *p* = 0.051). Geriatric nurse specialist certification was independently associated only with knowledge (*β* = 2.070, 95% CI 0.660 to 3.490; *p* = 0.004), not with overall KAP, attitude, or practice; sociodemographic characteristics were not associated.

**Conclusion:**

Nurses held positive attitudes, whereas self-reported practice corresponded to the lowest percentage of its maximum. Recency of frailty education was the factor most consistently associated with KAP and represents a potentially modifiable correlate. Given the cross-sectional design, these associations are consistent with a potential role for regularly refreshed, blended, and practice-oriented training. Longitudinal and interventional studies are needed to confirm clinical benefit.

## 1 Introduction

With the expanding global aging population, frailty has emerged as a critical public health challenge associated with increasing life expectancy (1). Frailty is a complex geriatric syndrome characterized by functional decline across multiple physiological systems and heightened vulnerability to stressors (2, 3). The global prevalence of pre-frailty and frailty among hospitalized patients is 39.3 and 29.8%, respectively (4), with rates of 41.3 and 22.6% reported in Chinese hospitalized older adults (5). More critically, frailty consistently predicts adverse clinical outcomes, including mortality, prolonged hospital stays, and functional deterioration (4). However, frailty is increasingly recognized as a dynamic and potentially reversible condition; emerging evidence suggests that with timely identification and targeted interventions, its progression can be slowed or even reversed (6), underscoring the importance of early detection and proactive management in clinical practice (1).

Among healthcare professionals, nurses play an important role in frailty management due to their sustained and direct patient contact. They are uniquely positioned to identify subtle indicators of functional decline, cognitive changes, and reduced physiological reserve, often before these changes are captured by formal assessment tools (7). Evidence demonstrates that nurse-led interventions can significantly improve functional outcomes and quality of life in pre-frail and frail older adults (8). Furthermore, targeted frailty education enhances nurses’ capabilities in both accurate assessment and effective intervention delivery, enabling them to implement comprehensive management strategies for frail older adults (7, 9). However, despite their frontline role, substantial variability exists in nurses’ frailty-related knowledge, attitudes, and practices—gaps that can compromise care quality and patient outcomes (10, 11). Therefore, a systematic assessment of nurses’ current competencies is essential to optimizing their contribution to frailty care.

Existing international evidence reveals concerning deficiencies in nurses’ frailty management competencies. Regarding knowledge, clinical nurses in Ireland predominantly relied on clinical judgment rather than formal training for frailty identification, resulting in poor knowledge levels and low confidence (12), while a Brazilian survey reported that fewer than 50% of healthcare professionals used standardized frailty assessment tools (13). In terms of attitudes, a Dutch study demonstrated that nearly all nurse respondents believed in the importance of preventive approaches to frailty and recognized their own role in preventing and reversing this condition (14). However, positive attitudes do not necessarily translate into appropriate practice; for instance, nurses in the Netherlands were found to be inappropriately using frailty screening tools despite their awareness of frailty’s significance (15). These findings collectively reveal substantial gaps between nurses’ knowledge, attitudes, and actual practice, underscoring the necessity of comprehensive assessment across these interrelated dimensions.

In China, Xuan and Liu (16) developed the first knowledge, attitude, and practice (KAP) questionnaire to assess nurses’ frailty management competencies based on the KAP framework, which posits that knowledge informs attitudes and that both shape practice (17); in reality, however, this progression is often incomplete, and the resulting knowledge–practice gap is itself worth characterizing. Subsequent studies have revealed concerning patterns: Huang et al. (18) identified marked discordance between knowledge and practice in a tertiary hospital in eastern China; Chen et al. (19) reported low-frequency implementation of frailty care practices among oncology nurses in southern China. More recently, Yang’s (20) mixed-methods study in northeastern China found that overall KAP levels were moderate, with the practice dimension scoring substantially lower than knowledge and attitude—a gap attributed to the absence of systematic training, insufficient managerial emphasis, and nursing workforce constraints. Moving beyond single-site designs, Chen et al. (21) conducted a multicenter cross-sectional survey of nurses in Fujian Province, southeastern China, and likewise found that nurses held relatively positive attitudes but that their overall KAP remained moderate, with practice again lagging behind knowledge and attitude. However, these studies share critical limitations: despite this recent shift toward multicenter designs, the evidence remains restricted to specialized units or coastal provinces, constraining generalizability, and nurses’ specific educational needs regarding frailty management remain unidentified. Most notably, existing evidence is concentrated in eastern, southern, and northeastern China, leaving southwestern China comparatively underexamined despite its comparably high aging burden. This omission is particularly consequential given the region’s disproportionate demographic pressures: according to China’s Seventh National Population Census, Chongqing Municipality and Sichuan Province recorded proportions of residents aged 65 years and over of 17.08 and 16.93%, respectively—exceeding those of most eastern and southern provinces (22). This demographic burden is compounded by higher frailty prevalence among older adults in southwestern China, potentially attributable to disparities in healthcare resources, economic development, and access to specialized geriatric services (23). Such regional disparities may, in turn, shape nurses’ frailty management competencies. Addressing these gaps requires extending the investigation to diverse clinical departments across multiple hospitals in southwestern China, coupled with a systematic assessment of training needs to inform context-specific educational programs.

In summary, frailty is highly prevalent among hospitalized older adults, nurses are central to its management, and addressing current gaps in frailty education is therefore *a priori*ty. The documented disconnect between knowledge and practice significantly constrains the quality of frailty care delivery. To develop evidence-based training interventions, a comprehensive understanding of nurses’ current competencies and their specific educational needs is essential—particularly in understudied regions such as southwestern China where the aging burden is most pronounced. To our knowledge, no multicenter study has jointly examined nurses’ frailty KAP, its correlates, and educational needs in southwestern China. Accordingly, to address these gaps and inform context-specific training, this study aimed to: (1) assess the levels of KAP regarding frailty among clinical nurses across multiple hospitals in southwestern China, and identify the sociodemographic and professional factors independently associated with these levels; (2) identify their educational needs to inform the development of future frailty training programs.

## 2 Methods

### 2.1 Ethical considerations

This study was conducted in accordance with the principles of the Declaration of Helsinki and was approved by the Institutional Review Board of The First Affiliated Hospital of Chongqing Medical University (approval number: 2024-151-01). The study involved human participants. Before the questionnaire could be accessed, all participants were presented with an information page describing the study purpose, procedures, anonymity, and their right to decline or to withdraw at any time without any consequence; provision of a response after reading this page constituted electronic informed consent. All responses were collected and stored anonymously and used solely for research purposes, and no personally identifiable information was recorded. Participation was entirely voluntary. To minimize any perceived pressure to participate, head nurses were asked only to forward the survey link and were explicitly instructed not to monitor, encourage, or record individual participation.

### 2.2 Study design

A cross-sectional survey design was employed utilizing anonymous questionnaire data collection. The research followed the Strengthening the Reporting of Observational Studies in Epidemiology (STROBE) reporting guidelines.

### 2.3 Participants and sample

Convenience sampling was employed for participant recruitment. An online questionnaire survey was conducted among nurses from 41 tertiary general hospitals in Chongqing Municipality and Sichuan Province between January and March 2025. These two regions are geographically proximate, share a common cultural heritage, and exhibit comparable levels of economic development. Inclusion criteria were as follows: (1) possession of a valid registered nurse license issued by the Chinese health authorities, (2) employed as full-time nurses, (3) clinical nursing experience exceeding 1 year, and (4) voluntary study participation. Exclusion criteria included: (1) nurses not affiliated with hospitals and (2) those on off-duty status during data collection.

The sample size was estimated using Kendall’s method, which recommends 5–10 participants per variable (24). With 55 variables, the required sample ranged from 275 to 550 participants. After inflating by 5% to allow for nonresponse or invalid questionnaires, the estimated requirement was 289 to 578 participants. We recognize that this participant-to-variable rule of thumb does not formally incorporate the number of parameters in the multivariable models or the clustering of nurses within hospitals; the achieved sample nonetheless provided a large number of observations per estimated regression parameter, the analyses were adjusted for within-hospital correlation (Section 2.6), and the associated reduction in effective sample size is acknowledged as a limitation. A total of 637 nurses were recruited, of whom 610 provided valid questionnaires, exceeding the estimated requirement.

### 2.4 Measurement

#### 2.4.1 Demographic data form

A sociodemographic questionnaire was developed to collect 13 self-reported characteristics: sex, age, years of nursing experience, highest education, professional title, position, employment status, department, participating hospital, geriatric nurse specialist certification, main work over the past year, recency of frailty education, and recency of frailty-related literature reading. Province was derived from the location of the participating hospital.

#### 2.4.2 Knowledge, attitude, and practice of frailty management questionnaire

The knowledge, attitude, and practice of frailty management questionnaire was developed using a systematic approach encompassing literature review, semi-structured interviews, initial instrument design, expert validation, pre-testing, and pilot evaluation. The final instrument comprised 31 items organized into three domains: knowledge, attitude, and practice.

The knowledge domain consisted of 14 items (score range: 14–42 points), including six subjective items and eight objective items. For subjective items, responses were scored as: “know” (3 points), “know a little” (2 points), or “do not know” (1 point). For objective items, responses were scored as: “correct” (3 points), “uncertain” (2 points), or “incorrect” (1 point). Higher scores indicated better knowledge of frailty management. The attitude domain comprised 8 items (score range: 8–40 points), rated on a 5-point Likert scale: 1 (strongly disagree), 2 (disagree), 3 (neutral), 4 (agree), and 5 (strongly agree). Higher scores indicated more positive attitudes toward frailty management. The practice domain consisted of 9 items (score range: 9–45 points), scored on a 5-point Likert scale: 1 (never), 2 (rarely), 3 (sometimes), 4 (often), and 5 (always). Higher scores indicated more frequent engagement in frailty management practices. The total KAP score ranged from 31 to 127 points, with higher scores representing superior knowledge, attitudes, and practices regarding frailty management (Supplementary Table 1).

Raw scores are reported as the primary metric. For descriptive purposes only, each domain and the total were additionally expressed as a percentage of the maximum possible score [(observed score / maximum possible score) × 100%]. Because the domains have non-zero minimum scores and differ in the number of items and response ranges, these percentages have unequal floors and are not directly comparable across domains; they are presented only to aid interpretation.

The original questionnaire demonstrated excellent psychometric properties, with test–retest reliability (*r* = 0.990) and Cronbach’s *α* coefficients of 0.909 for the overall scale (16). In the present study, Cronbach’s *α* coefficients calculated on the full analytic sample (*n* = 610) also indicated good internal consistency, with values of 0.879 for the knowledge domain, 0.929 for the attitude domain, 0.949 for the practice domain, and 0.930 for the total scale.

#### 2.4.3 Educational needs of frailty questionnaire

Educational needs were assessed with an 11-item questionnaire developed by the research team on the basis of a literature review and group discussion, covering preferred training content, training formats, and teaching methods. The first item asked whether the respondent was willing to participate in frailty training; respondents who answered “yes” completed the remaining items, whereas those who answered “no” did not proceed. The remaining items used single-choice items (e.g., preferred training format and session duration) and multiple-choice items (e.g., preferred content and teaching methods), for which respondents could select all applicable options. Because these items capture multiple-choice training preferences rather than a latent construct, internal-consistency and factor-analytic indices are not applicable, and this section was summarized descriptively.

### 2.5 Data collection and quality control

Data were collected through an electronic questionnaire developed on the Questionnaire Star platform (Changsha Ranxing Information Technology Co., Ltd., Changsha, China) and distributed via WeChat (Tencent Holdings Ltd., Shenzhen, China), a major Chinese social media platform, using both direct URL links and Quick Response codes. The research team coordinated with head nurses from each participating unit to explain the study’s purpose and methodology prior to distribution. To ensure data integrity and prevent duplicate submissions, each IP address was restricted to one questionnaire completion, and all questionnaire items were configured as mandatory fields. Participants completed the questionnaires anonymously, and the resulting data were directly exported from the platform for analysis. The mandatory-response design eliminated item-level missing data. To maintain data validity, two prespecified quality-control rules were applied. First, questionnaires completed in less than 3 min were excluded; this threshold was derived from pilot testing, in which the shortest plausible time for an attentive respondent to read and answer all items exceeded 3 min, so shorter completions were considered indicative of inattentive or non-genuine responding. Second, questionnaires exhibiting straight-line responding were excluded; this was defined *a priori* as consistently selecting the same response option (e.g., always the first or the last choice) across all items. Two researchers independently screened records against this criterion, and disagreements were resolved by discussion.

### 2.6 Statistical analysis

All analyses were performed in R (version 4.4.0). Continuous variables were summarized as mean ± standard deviation (SD) and categorical variables as frequencies (percentages); educational needs were summarized descriptively among nurses willing to receive training.

Because nurses were nested within hospitals, associations were assessed using linear mixed-effects models with a random intercept for hospital. For each outcome (the total KAP score and the knowledge, attitude, and practice subscales), a null model was first fitted to estimate the ICC. Single-variable models were used for screening, and a single multivariable model per outcome was then specified *a priori*, entering 11 clinically and theoretically relevant covariates simultaneously (age, sex, province, years of nursing experience, highest education, professional title, department, geriatric nurse specialist certification, main work over the past year, recency of frailty education, and recency of frailty-related literature reading). Covariate selection was thus driven by an *a priori* clinical strategy rather than by univariate *p*-values. Of the candidate variables, position and employment status were examined in the single-variable analyses but were not entered into the multivariable models: administrative position (staff vs. head nurse) is closely correlated with professional title and would have introduced collinearity, whereas employment status was not a prespecified variable of primary interest and was omitted to conserve degrees of freedom. Province and department were retained as contextual covariates.

Models were fitted by restricted maximum likelihood, and fixed effects were tested with Type III *F*-tests using the Satterthwaite approximation. For each variable an overall *p* value is reported, and for each non-reference category the *β* coefficient with its 95% confidence interval (CI). Multicollinearity was assessed by the variance inflation factor (all VIF < 10 except the two correlated primary exposures, which were retained); residual-versus-fitted and normal quantile–quantile plots were inspected and judged acceptable; and model performance was summarized by the marginal *R*^2^. Given the exploratory objectives, all *p*-values are interpreted descriptively, category-level contrasts are regarded as hypothesis-generating, and all tests were two-sided with *p* < 0.05 considered significant.

## 3 Results

### 3.1 Sample characteristics and data collection

A total of 637 questionnaires were collected. After excluding 27 questionnaires (12 questionnaires were excluded because of a completion time of <3 min, and 15 were excluded due to straight-line responding), 610 valid questionnaires were retained for analysis; the proportion of valid questionnaires was 95.76% (610/637). Because the number of nurses who received the survey link could not be determined, this proportion reflects the valid-questionnaire rate and should not be interpreted as a true response rate.

Respondents were drawn from 41 hospitals across Chongqing Municipality (531/610, 87.05%) and Sichuan Province (79/610, 12.95%). The sample was predominantly female (591/610, 96.89%). Respondents were distributed across a wide range of departments, most commonly internal-medicine departments (259/610, 42.46%). Full participant characteristics, together with mean scores across subgroups, are presented in Table 1; the distribution of respondents by hospital and by department is provided in Supplementary Tables 2, 3.

**Table 1:** Participant characteristics and knowledge, attitude, and practice scores across subgroups (*n* = 610).

Characteristic	Category	*n* (%)	Total KAP score	Knowledge	Attitude	Practice
Sex	Male	19 (3.11%)	101.84 ± 13.09	34.47 ± 4.01	34.74 ± 4.87	32.63 ± 7.80
	Female	591 (96.89%)	103.11 ± 12.64	34.17 ± 4.20	35.43 ± 4.59	33.51 ± 7.47
Age group (years)	≤25	24 (3.93%)	102.67 ± 13.53	35.13 ± 6.18	35.21 ± 5.46	32.33 ± 8.19
	26–35	315 (51.64%)	102.43 ± 12.71	33.88 ± 4.08	35.24 ± 4.77	33.31 ± 7.25
	>35	271 (44.43%)	103.85 ± 12.50	34.45 ± 4.10	35.62 ± 4.31	33.78 ± 7.68
Nursing experience (years)	≤3	62 (10.16%)	101.74 ± 11.91	34.50 ± 4.65	34.53 ± 4.81	32.71 ± 6.91
	4–5	41 (6.72%)	104.85 ± 12.89	34.41 ± 4.23	35.98 ± 5.28	34.46 ± 6.61
	6–10	140 (22.95%)	101.63 ± 12.15	33.66 ± 3.58	35.24 ± 4.63	32.73 ± 7.84
	11–15	206 (33.77%)	103.39 ± 13.40	34.12 ± 4.61	35.81 ± 4.56	33.47 ± 7.55
	>15	161 (26.39%)	103.98 ± 12.28	34.52 ± 3.92	35.25 ± 4.33	34.20 ± 7.47
Province	Chongqing	531 (87.05%)	102.75 ± 12.80	34.04 ± 4.28	35.40 ± 4.65	33.31 ± 7.59
	Sichuan	79 (12.95%)	105.23 ± 11.43	35.13 ± 3.39	35.48 ± 4.27	34.62 ± 6.62
Highest education	Master’s degree or above	16 (2.62%)	102.81 ± 9.31	35.56 ± 3.81	36.19 ± 3.66	31.06 ± 6.72
	Bachelor’s degree	535 (87.70%)	103.06 ± 12.74	34.10 ± 4.14	35.42 ± 4.62	33.54 ± 7.52
	Associate degree	58 (9.51%)	103.43 ± 12.81	34.59 ± 4.77	35.22 ± 4.62	33.62 ± 7.35
	Technical secondary school	1 (0.16%)	94.00 (—)	30.00 (—)	31.00 (—)	33.00 (—)
Department	Internal medicine	259 (42.46%)	102.81 ± 12.17	34.05 ± 3.86	35.12 ± 4.66	33.64 ± 7.39
	Geriatrics/general practice/health management	85 (13.93%)	105.84 ± 11.38	35.05 ± 4.39	35.87 ± 4.08	34.92 ± 6.50
	Oncology	59 (9.67%)	102.88 ± 13.78	34.15 ± 4.10	35.20 ± 4.57	33.53 ± 7.98
	Surgical	61 (10.00%)	104.07 ± 12.65	34.13 ± 4.81	35.21 ± 4.74	34.72 ± 5.99
	Obstetrics and gynecology	23 (3.77%)	100.39 ± 13.75	32.52 ± 4.01	35.26 ± 5.19	32.61 ± 7.61
	Emergency and critical care	19 (3.11%)	101.53 ± 12.31	34.16 ± 3.59	34.84 ± 5.08	32.53 ± 5.83
	Ophthalmology, ENT and stomatology	19 (3.11%)	102.26 ± 10.66	32.95 ± 3.89	36.47 ± 4.18	32.84 ± 6.39
	Nursing administration	8 (1.31%)	101.13 ± 15.18	36.13 ± 4.76	34.63 ± 5.34	30.38 ± 10.10
	Other	77 (12.62%)	101.83 ± 14.52	34.32 ± 4.69	36.19 ± 4.59	31.31 ± 9.24
Professional title	Chief nurse	8 (1.31%)	103.13 ± 14.90	37.63 ± 3.50	34.75 ± 4.56	30.75 ± 8.43
	Associate chief nurse	55 (9.02%)	104.84 ± 12.37	35.05 ± 4.07	35.24 ± 4.18	34.55 ± 6.48
	Supervising nurse	274 (44.92%)	103.34 ± 12.84	34.00 ± 4.39	35.75 ± 4.54	33.58 ± 7.55
	Senior nurse	217 (35.57%)	102.49 ± 12.51	33.94 ± 3.72	35.27 ± 4.71	33.27 ± 7.72
	Nurse	56 (9.18%)	102.30 ± 12.45	34.61 ± 4.88	34.55 ± 4.82	33.14 ± 7.02
Position	Staff nurse	527 (86.39%)	102.77 ± 12.75	33.93 ± 4.17	35.38 ± 4.66	33.46 ± 7.55
	Director/deputy director of nursing	6 (0.98%)	102.67 ± 12.47	37.17 ± 3.82	34.67 ± 5.92	30.83 ± 4.75
	Head nurse	77 (12.62%)	105.18 ± 11.90	35.62 ± 4.06	35.70 ± 4.06	33.86 ± 7.15
Employment status	Contract staff	394 (64.59%)	102.95 ± 13.32	34.10 ± 4.19	35.37 ± 4.83	33.48 ± 7.90
	Permanent staff	216 (35.41%)	103.30 ± 11.35	34.32 ± 4.20	35.50 ± 4.14	33.49 ± 6.65
Geriatric nurse specialist certification	No	577 (94.59%)	102.80 ± 12.61	34.00 ± 4.17	35.36 ± 4.64	33.44 ± 7.44
	Yes	33 (5.41%)	107.79 ± 12.52	37.24 ± 3.48	36.33 ± 3.67	34.21 ± 8.21
Main work over the past year	Non-clinical nursing	61 (10.00%)	105.95 ± 11.40	35.62 ± 4.25	36.34 ± 4.29	33.98 ± 6.47
	Clinical nursing	549 (90.00%)	102.75 ± 12.75	34.02 ± 4.16	35.31 ± 4.62	33.43 ± 7.59
Recency of frailty education (months)	<1	36 (5.90%)	108.72 ± 13.61	36.11 ± 4.73	36.81 ± 3.62	35.81 ± 8.80
	1–6	80 (13.11%)	110.55 ± 10.24	36.89 ± 3.83	37.14 ± 3.61	36.53 ± 6.55
	7–12	53 (8.69%)	108.11 ± 10.31	35.70 ± 4.02	35.83 ± 3.70	36.58 ± 5.61
	13–24	66 (10.82%)	105.82 ± 11.31	35.65 ± 3.26	35.50 ± 4.69	34.67 ± 6.61
	>24	61 (10.00%)	104.26 ± 13.79	35.36 ± 3.57	35.11 ± 4.48	33.79 ± 8.39
	Never	314 (51.48%)	98.86 ± 11.89	32.47 ± 3.79	34.78 ± 4.92	31.61 ± 7.28
Recency of frailty-related literature reading (months)	<1	45 (7.38%)	107.53 ± 13.06	36.02 ± 4.91	37.04 ± 3.30	34.47 ± 8.90
	1–6	97 (15.90%)	110.45 ± 9.94	36.68 ± 3.80	37.13 ± 3.70	36.64 ± 5.94
	7–12	54 (8.85%)	107.15 ± 10.48	35.37 ± 4.09	35.50 ± 4.01	36.28 ± 5.46
	13–24	61 (10.00%)	104.85 ± 10.72	35.36 ± 2.97	35.57 ± 4.31	33.92 ± 6.30
	>24	73 (11.97%)	103.88 ± 12.79	34.77 ± 3.16	34.92 ± 4.42	34.19 ± 7.87
	Never	280 (45.90%)	98.41 ± 12.35	32.38 ± 3.91	34.63 ± 5.05	31.41 ± 7.59

Values are mean ± SD unless otherwise indicated. “(—)” denotes that SD is not applicable (subgroup *n* = 1). KAP, knowledge, attitude, and practice.

### 3.2 Knowledge, attitude, and practice scores regarding frailty

As shown in Table 2, nurses demonstrated moderately high levels of knowledge, attitude, and practice regarding frailty, with an overall mean score of 103.07 ± 12.65 (81.16% of the total possible score). Among the three dimensions, attitude achieved the highest performance (35.41 ± 4.60, 88.53%), followed by knowledge (34.18 ± 4.19, 81.38%) and practice (33.48 ± 7.48, 74.40% of the maximum). Because the domains use different scales and have unequal score floors, these percentages are not strictly comparable across domains; nevertheless, practice corresponded to the lowest percentage of its own maximum, a pattern consistent with prior reports of practice lagging behind knowledge and attitude.

**Table 2:** Descriptive statistics of knowledge, attitude, and practice scores among nurses (*n* = 610).

Dimension	Min	Max	Mean (SD)	Score rate (%)
Knowledge	14	42	34.18 (4.19)	81.38
Attitude	21	40	35.41 (4.60)	88.53
Practice	9	45	33.48 (7.48)	74.40
Total	60	127	103.07 (12.65)	81.16

Minimum and maximum are the observed values. Score rate (%) = mean/maximum possible score × 100. SD, standard deviation.

### 3.3 Between-hospital clustering

In the intercept-only models, the ICCs were 0.036 for the total KAP score, 0.067 for knowledge, 0.008 for attitude, and 0.037 for practice, indicating that only 3.6, 6.7, 0.8, and 3.7% of the total variance in each score, respectively, was attributable to differences between hospitals. Although this clustering was small, all subsequent models retained a random intercept for hospital to account for the hierarchical structure of the data.

### 3.4 Univariate mixed-effects analyses

Single-variable mixed-effects models were used to examine associations between participant characteristics and each score (Table 3). The total KAP score was associated with geriatric nurse specialist certification (*β* = 4.500, 95% CI 0.050 to 8.940; *p* = 0.047), recency of frailty education (overall *p* < 0.001), and recency of frailty-related literature reading (overall *p* < 0.001). Both exposure variables followed a graded pattern: relative to nurses with the most recent exposure (<1 month), those who had never received training scored markedly lower (*β* = −9.830, 95% CI − 13.900 to −5.760; *p* < 0.001), as did those who had never read frailty-related literature (*β* = −9.210, 95% CI − 12.930 to −5.500; *p* < 0.001).

**Table 3:** Univariate linear mixed-effects models of factors associated with frailty-management knowledge, attitude, and practice scores (*n* = 610).

Characteristic	Total KAP score		Knowledge		Attitude		Practice	
	*β* (95% CI)	*p*	*β* (95% CI)	*p*	*β* (95% CI)	*p*	*β* (95% CI)	*p*
**Sex**		0.850		0.578		0.550		0.812
Female	Reference		Reference		Reference		Reference	
Male	−0.560 (−6.300 to 5.190)	0.850	0.540 (−1.360 to 2.430)	0.578	−0.640 (−2.740 to 1.460)	0.550	−0.410 (−3.810 to 2.990)	0.812
**Age (per year)**	0.030 (−0.110 to 0.180)	0.650	0.010 (−0.040 to 0.060)	0.671	0.000 (−0.050 to 0.050)	0.989	0.020 (−0.060 to 0.110)	0.588
**Nursing experience (per year)**	0.100 (−0.040 to 0.240)	0.146	0.030 (−0.010 to 0.080)	0.157	0.010 (−0.040 to 0.060)	0.612	0.060 (−0.020 to 0.140)	0.165
**Province**		0.591		0.441		0.768		0.531
Chongqing	Reference		Reference		Reference		Reference	
Sichuan	1.000 (−2.740 to 4.730)	0.591	0.520 (−0.840 to 1.880)	0.441	−0.180 (−1.380 to 1.030)	0.768	0.690 (−1.520 to 2.900)	0.531
**Highest education**		0.858		0.169		0.584		0.766
Bachelor’s degree	Reference		Reference		Reference		Reference	
Master’s degree or above	1.050 (−5.250 to 7.350)	0.744	1.930 (−0.130 to 4.000)	0.066	1.040 (−1.260 to 3.350)	0.373	−2.000 (−5.730 to 1.720)	0.292
Associate degree	0.040 (−3.400 to 3.480)	0.980	0.420 (−0.720 to 1.550)	0.470	−0.230 (−1.480 to 1.020)	0.722	−0.170 (−2.210 to 1.860)	0.869
Technical secondary school	−10.320 (−35.380 to 14.740)	0.419	−4.700 (−13.060 to 3.660)	0.270	−4.610 (−13.670 to 4.450)	0.318	−1.040 (−15.860 to 13.770)	0.890
**Department**		0.681		0.522		0.756		0.112
Internal medicine	Reference		Reference		Reference		Reference	
Geriatrics/general practice/health management	1.980 (−1.330 to 5.300)	0.241	0.580 (−0.540 to 1.690)	0.312	0.680 (−0.470 to 1.830)	0.245	0.810 (−1.120 to 2.740)	0.411
Oncology	1.040 (−2.590 to 4.660)	0.574	0.310 (−0.890 to 1.500)	0.613	0.150 (−1.170 to 1.460)	0.826	0.440 (−1.690 to 2.570)	0.686
Surgical	0.030 (−3.680 to 3.730)	0.989	−0.240 (−1.480 to 1.000)	0.704	0.010 (−1.300 to 1.320)	0.989	0.440 (−1.730 to 2.610)	0.690
Obstetrics and gynecology	−2.420 (−7.810 to 2.960)	0.377	−1.460 (−3.230 to 0.320)	0.108	0.130 (−1.830 to 2.100)	0.894	−1.080 (−4.250 to 2.080)	0.501
Emergency and critical care	−1.380 (−7.270 to 4.510)	0.645	0.270 (−1.670 to 2.210)	0.786	−0.300 (−2.450 to 1.860)	0.787	−1.300 (−4.770 to 2.160)	0.461
Ophthalmology, ENT and stomatology	−0.060 (−5.930 to 5.820)	0.985	−0.950 (−2.890 to 0.980)	0.334	1.380 (−0.770 to 3.530)	0.208	−0.540 (−4.000 to 2.920)	0.760
Nursing administration	−3.160 (−12.220 to 5.900)	0.493	1.740 (−1.280 to 4.760)	0.259	−0.590 (−3.850 to 2.670)	0.721	−4.140 (−9.450 to 1.170)	0.127
Other	−2.240 (−5.940 to 1.460)	0.234	−0.120 (−1.380 to 1.140)	0.851	0.950 (−0.290 to 2.190)	0.131	−2.820 (−4.960 to −0.670)	**0.010**
**Professional title**		0.958		0.139		0.493		0.746
Supervising nurse	Reference		Reference		Reference		Reference	
Chief nurse	−0.900 (−9.810 to 8.000)	0.842	3.270 (0.350 to 6.200)	**0.028**	−1.090 (−4.330 to 2.150)	0.509	−3.080 (−8.340 to 2.180)	0.251
Associate chief nurse	0.920 (−2.810 to 4.650)	0.628	0.820 (−0.420 to 2.050)	0.193	−0.570 (−1.920 to 0.770)	0.400	0.680 (−1.520 to 2.890)	0.544
Senior nurse	−0.420 (−2.720 to 1.880)	0.718	0.020 (−0.740 to 0.780)	0.958	−0.390 (−1.230 to 0.440)	0.351	−0.110 (−1.470 to 1.250)	0.872
Nurse	−0.730 (−4.400 to 2.940)	0.698	0.610 (−0.590 to 1.820)	0.318	−1.110 (−2.440 to 0.220)	0.101	−0.320 (−2.490 to 1.850)	0.769
**Position**		0.519		**0.005**		0.861		0.582
Staff nurse	Reference		Reference		Reference		Reference	
Director/deputy director of nursing	−0.810 (−11.050 to 9.430)	0.876	3.090 (−0.280 to 6.460)	0.073	−0.800 (−4.520 to 2.910)	0.672	−3.120 (−9.180 to 2.940)	0.312
Head nurse	1.770 (−1.320 to 4.850)	0.261	1.460 (0.450 to 2.470)	**0.005**	0.190 (−0.930 to 1.300)	0.744	0.180 (−1.650 to 2.000)	0.848
**Employment status**		0.830		0.561		0.682		0.763
Contract staff	Reference		Reference		Reference		Reference	
Permanent staff	0.230 (−1.900 to 2.370)	0.830	0.210 (−0.500 to 0.920)	0.561	0.160 (−0.610 to 0.930)	0.682	−0.190 (−1.460 to 1.070)	0.763
**Geriatric nurse specialist certification**		**0.047**		**<0.001**		0.258		0.741
No	Reference		Reference		Reference		Reference	
Yes	4.500 (0.050 to 8.940)	**0.047**	3.080 (1.620 to 4.540)	**<0.001**	0.930 (−0.690 to 2.550)	0.258	0.440 (−2.190 to 3.080)	0.741
**Main work over the past year**		0.146		**0.029**		0.133		0.712
Clinical nursing	Reference		Reference		Reference		Reference	
Non-clinical nursing	2.540 (−0.880 to 5.960)	0.146	1.260 (0.130 to 2.400)	**0.029**	0.940 (−0.290 to 2.170)	0.133	0.380 (−1.650 to 2.410)	0.712
**Recency of frailty education (months)**		**<0.001**		**<0.001**		**<0.001**		**<0.001**
<1	Reference		Reference		Reference		Reference	
1–6	1.660 (−3.000 to 6.330)	0.484	0.810 (−0.690 to 2.300)	0.291	0.330 (−1.460 to 2.120)	0.717	0.460 (−2.380 to 3.300)	0.752
7–12	−0.890 (−5.890 to 4.120)	0.727	−0.420 (−2.030 to 1.180)	0.605	−0.990 (−2.920 to 0.930)	0.311	0.460 (−2.590 to 3.510)	0.766
13–24	−2.960 (−7.760 to 1.830)	0.225	−0.470 (−2.010 to 1.070)	0.550	−1.300 (−3.140 to 0.540)	0.167	−1.240 (−4.160 to 1.680)	0.405
>24	−4.680 (−9.550 to 0.180)	0.059	−0.770 (−2.330 to 0.780)	0.330	−1.700 (−3.570 to 0.170)	0.074	−2.280 (−5.240 to 0.680)	0.131
Never	−9.830 (−13.900 to −5.760)	**<0.001**	−3.560 (−4.870 to −2.260)	**<0.001**	−2.000 (−3.560 to −0.430)	**0.013**	−4.320 (−6.800 to −1.840)	**<0.001**
**Recency of frailty-related literature reading (months)**		**<0.001**		**<0.001**		**<0.001**		**<0.001**
<1	Reference		Reference		Reference		Reference	
1–6	2.640 (−1.540 to 6.820)	0.215	0.550 (−0.800 to 1.910)	0.422	0.090 (−1.510 to 1.680)	0.915	1.930 (−0.620 to 4.470)	0.138
7–12	−0.660 (−5.320 to 4.000)	0.781	−0.660 (−2.170 to 0.850)	0.393	−1.550 (−3.330 to 0.240)	0.089	1.490 (−1.350 to 4.330)	0.304
13–24	−2.960 (−7.490 to 1.580)	0.201	−0.740 (−2.210 to 0.740)	0.326	−1.470 (−3.210 to 0.270)	0.097	−0.830 (−3.590 to 1.940)	0.558
>24	−3.800 (−8.180 to 0.590)	0.090	−1.290 (−2.710 to 0.130)	0.076	−2.110 (−3.790 to −0.440)	**0.014**	−0.490 (−3.160 to 2.190)	0.722
Never	−9.210 (−12.930 to −5.500)	**<0.001**	−3.630 (−4.840 to −2.430)	**<0.001**	−2.400 (−3.820 to −0.980)	**<0.001**	−3.280 (−5.550 to −1.010)	**0.005**

Each model included a random intercept for hospital. For categorical variables, the overall *p* value (variable-level Type III test) is shown on the variable row and estimates are relative to the reference category. *β*, unstandardized regression coefficient; CI, confidence interval. Statistically significant values (*p* < 0.05) are shown in bold.

The knowledge score was associated with the largest number of characteristics, including geriatric nurse specialist certification (*β* = 3.080, 95% CI 1.620 to 4.540; *p* < 0.001), administrative position (overall *p* = 0.005; head nurses scored higher than staff nurses: *β* = 1.460, 95% CI 0.450 to 2.470), main work over the past year (overall *p* = 0.029), and both exposure variables (both overall *p* < 0.001). The attitude and practice scores were each associated only with recency of frailty education and recency of literature reading (all overall *p* < 0.001), again following a graded pattern (for practice, never vs. < 1 month of training: *β* = −4.320, 95% CI − 6.800 to −1.840; *p* < 0.001). Sex, age, years of nursing experience, province, education, professional title, and employment status were not significantly associated with the total score (all overall *p* > 0.05).

### 3.5 Multivariable mixed-effects analyses

In the multivariable models, each of which included all 11 *a priori* covariates and a random intercept for hospital, associations were attenuated relative to the univariate analyses (Table 4). For the total KAP score (marginal *R*^2^ = 0.171), recency of frailty education remained associated overall (*p* = 0.019), with never-trained nurses scoring lower than recently trained nurses (*β* = −6.820, 95% CI − 13.220 to −0.430; *p* = 0.036); the overall association for recency of reading was borderline (*p* = 0.051). For the knowledge score (marginal *R*^2^ = 0.234), geriatric nurse specialist certification was independently associated with higher scores (*β* = 2.070, 95% CI 0.660 to 3.490; *p* = 0.004), and recency of frailty education retained a strong overall association (*p* < 0.001), although individual category contrasts were attenuated after adjustment; recency of reading was also associated overall (*p* = 0.035). For the attitude score (marginal *R*^2^ = 0.068), no covariate reached overall significance, including recency of education (*p* = 0.858) and recency of reading (*p* = 0.338). For the practice score (marginal *R*^2^ = 0.126), department was associated overall (*p* = 0.037), and never-trained nurses scored lower than recently trained nurses (*β* = −4.360, 95% CI − 8.250 to −0.480; *p* = 0.028), although the overall association for recency of education did not reach significance (*p* = 0.096). Consistent with an exploratory approach, several category-level contrasts reached nominal significance while the corresponding overall variable test did not (e.g., the “other” department category for the total score); these isolated contrasts are interpreted as hypothesis-generating.

**Table 4:** Multivariable linear mixed-effects models of factors associated with frailty-management knowledge, attitude, and practice scores (*n* = 610).

Characteristic	Total KAP score		Knowledge		Attitude		Practice	
	*β* (95% CI)	*p*	*β* (95% CI)	*p*	*β* (95% CI)	*p*	*β* (95% CI)	*p*
**Age (per year)**	−0.200 (−0.500 to 0.100)	0.190	−0.050 (−0.150 to 0.040)	0.273	−0.040 (−0.160 to 0.080)	0.502	−0.110 (−0.290 to 0.080)	0.258
**Sex**		0.883		0.621		0.613		0.869
Female	Reference		Reference		Reference		Reference	
Male	−0.420 (−6.010 to 5.170)	0.883	0.450 (−1.320 to 2.220)	0.621	−0.560 (−2.720 to 1.610)	0.613	−0.290 (−3.680 to 3.110)	0.869
**Province**		0.131		0.117		0.641		0.205
Chongqing	Reference		Reference		Reference		Reference	
Sichuan	2.520 (−0.780 to 5.810)	0.131	0.890 (−0.230 to 2.010)	0.117	0.280 (−0.910 to 1.480)	0.641	1.310 (−0.730 to 3.360)	0.205
**Nursing experience (per year)**	0.280 (−0.040 to 0.600)	0.083	0.070 (−0.030 to 0.170)	0.157	0.040 (−0.080 to 0.170)	0.486	0.160 (−0.030 to 0.360)	0.096
**Highest education**		0.846		0.460		0.694		0.637
Bachelor’s degree	Reference		Reference		Reference		Reference	
Master’s degree or above	−0.630 (−6.770 to 5.500)	0.840	1.190 (−0.760 to 3.140)	0.230	0.570 (−1.790 to 2.940)	0.634	−2.370 (−6.100 to 1.360)	0.212
Associate degree	0.010 (−3.530 to 3.560)	0.994	0.180 (−0.950 to 1.310)	0.753	0.170 (−1.200 to 1.530)	0.813	−0.320 (−2.480 to 1.840)	0.770
Technical secondary school	−10.610 (−34.370 to 13.140)	0.381	−3.880 (−11.450 to 3.700)	0.315	−4.990 (−14.170 to 4.190)	0.286	−1.740 (−16.190 to 12.720)	0.814
**Professional title**		0.695		0.574		0.164		0.592
Supervising nurse	Reference		Reference		Reference		Reference	
Chief nurse	−5.070 (−14.860 to 4.710)	0.309	1.660 (−1.450 to 4.760)	0.295	−2.120 (−5.900 to 1.670)	0.272	−4.600 (−10.540 to 1.340)	0.129
Associate chief nurse	−2.170 (−6.500 to 2.150)	0.324	−0.210 (−1.590 to 1.170)	0.767	−1.370 (−3.040 to 0.300)	0.107	−0.620 (−3.250 to 2.010)	0.645
Senior nurse	−0.520 (−2.940 to 1.900)	0.674	−0.010 (−0.780 to 0.770)	0.989	−0.380 (−1.310 to 0.540)	0.415	−0.130 (−1.600 to 1.350)	0.868
Nurse	−1.360 (−5.710 to 2.990)	0.540	0.710 (−0.680 to 2.100)	0.316	−1.370 (−3.040 to 0.300)	0.107	−0.680 (−3.330 to 1.970)	0.614
**Department**		0.281		0.211		0.843		**0.037**
Internal medicine	Reference		Reference		Reference		Reference	
Surgical	−0.110 (−3.520 to 3.300)	0.948	−0.240 (−1.340 to 0.860)	0.667	−0.200 (−1.500 to 1.100)	0.764	0.320 (−1.760 to 2.410)	0.760
Emergency and critical care	−1.550 (−7.210 to 4.120)	0.592	0.070 (−1.730 to 1.870)	0.936	−0.200 (−2.400 to 1.990)	0.855	−1.360 (−4.810 to 2.080)	0.436
Nursing administration	−4.820 (−14.040 to 4.400)	0.305	−0.450 (−3.390 to 2.500)	0.766	−0.760 (−4.320 to 2.800)	0.674	−3.590 (−9.200 to 2.020)	0.210
Other	−3.620 (−6.970 to −0.280)	**0.034**	−0.410 (−1.520 to 0.690)	0.458	0.450 (−0.780 to 1.670)	0.474	−3.550 (−5.610 to −1.480)	**<0.001**
Geriatrics/general practice/health management	−0.430 (−3.640 to 2.780)	0.793	−0.680 (−1.730 to 0.360)	0.197	0.280 (−0.930 to 1.490)	0.650	−0.040 (−2.010 to 1.930)	0.968
Obstetrics and gynecology	−4.700 (−9.870 to 0.470)	0.075	−2.410 (−4.050 to −0.770)	**0.004**	0.030 (−1.980 to 2.030)	0.980	−2.280 (−5.420 to 0.860)	0.155
Oncology	0.870 (−2.580 to 4.310)	0.621	0.380 (−0.720 to 1.480)	0.496	0.210 (−1.120 to 1.530)	0.759	0.300 (−1.790 to 2.400)	0.775
Ophthalmology, ENT and stomatology	2.130 (−3.450 to 7.700)	0.454	−0.420 (−2.180 to 1.350)	0.645	1.880 (−0.270 to 4.040)	0.087	0.690 (−2.700 to 4.070)	0.690
**Geriatric nurse specialist certification**		0.688		**0.004**		0.745		0.290
No	Reference		Reference		Reference		Reference	
Yes	0.910 (−3.540 to 5.360)	0.688	2.070 (0.660 to 3.490)	**0.004**	0.290 (−1.430 to 2.000)	0.745	−1.460 (−4.160 to 1.250)	0.290
**Main work over the past year**		0.273		0.407		0.187		0.623
Clinical nursing	Reference		Reference		Reference		Reference	
Non-clinical nursing	1.980 (−1.560 to 5.530)	0.273	0.480 (−0.650 to 1.610)	0.407	0.920 (−0.450 to 2.290)	0.187	0.540 (−1.620 to 2.700)	0.623
**Recency of frailty education (months)**		**0.019**		**<0.001**		0.858		0.096
<1	Reference		Reference		Reference		Reference	
1–6	0.060 (−6.030 to 6.160)	0.984	1.120 (−0.810 to 3.060)	0.255	0.330 (−2.030 to 2.690)	0.783	−1.390 (−5.090 to 2.310)	0.461
7–12	−0.650 (−7.530 to 6.220)	0.853	0.470 (−1.720 to 2.650)	0.675	−0.060 (−2.720 to 2.610)	0.966	−1.070 (−5.250 to 3.110)	0.615
13–24	−2.850 (−9.620 to 3.930)	0.409	0.460 (−1.690 to 2.610)	0.675	−0.590 (−3.220 to 2.030)	0.657	−2.700 (−6.820 to 1.410)	0.198
>24	−3.690 (−10.840 to 3.460)	0.311	0.270 (−2.000 to 2.540)	0.815	−0.570 (−3.340 to 2.200)	0.686	−3.410 (−7.750 to 0.940)	0.124
Never	−6.820 (−13.220 to −0.430)	**0.036**	−1.690 (−3.720 to 0.340)	0.102	−0.760 (−3.240 to 1.710)	0.546	−4.360 (−8.250 to −0.480)	**0.028**
**Recency of frailty-related literature reading (months)**		0.051		**0.035**		0.338		0.165
<1	Reference		Reference		Reference		Reference	
1–6	1.930 (−3.450 to 7.320)	0.481	0.000 (−1.710 to 1.700)	0.995	−0.200 (−2.290 to 1.880)	0.850	2.120 (−1.150 to 5.390)	0.204
7–12	−1.000 (−7.350 to 5.340)	0.756	−1.030 (−3.040 to 0.980)	0.315	−1.610 (−4.070 to 0.850)	0.199	1.630 (−2.220 to 5.480)	0.406
13–24	−1.150 (−7.430 to 5.140)	0.720	−0.510 (−2.510 to 1.480)	0.613	−1.150 (−3.590 to 1.280)	**0.352**	0.510 (−3.310 to 4.320)	0.794
>24	−1.310 (−7.710 to 5.100)	0.689	−0.990 (−3.020 to 1.050)	0.342	−1.710 (−4.190 to 0.770)	0.176	1.390 (−2.500 to 5.290)	0.482
Never	−4.620 (−10.420 to 1.180)	0.118	−1.980 (−3.820 to −0.140)	**0.035**	−1.850 (−4.090 to 0.400)	0.107	−0.800 (−4.330 to 2.720)	0.655

Each model included all 11 *a priori* covariates and a random intercept for hospital. Overall *p*-values (variable-level Type III tests) are shown on the variable row; estimates are relative to the reference category. Marginal *R*^2^ = 0.171 (total KAP), 0.234 (knowledge), 0.068 (attitude), and 0.126 (practice). *β*, unstandardized regression coefficient; CI, confidence interval. Statistically significant values (*p* < 0.05) are shown in bold.

### 3.6 Frailty educational needs

Of the 610 respondents, 558 (91.48%) expressed willingness to participate in frailty-management training, whereas 52 (8.52%) did not; the educational-needs analysis was restricted to the 558 willing participants (Table 5). The most frequently endorsed theoretical topics were frailty prevention and intervention (both 89.61%), risk factors (87.99%), and assessment (87.63%). For practical skills, health education (91.22%), nutritional intervention (91.04%), and exercise intervention (90.86%) were most frequently selected, and 92.47% endorsed learning about the feasibility and effectiveness of early intervention. A blended online–offline format was preferred by 66.85% of respondents, well above purely online (24.19%) or face-to-face (8.96%) formats. The most favored learning channels were online information dissemination and academic conferences (both 76.52%), followed by literature sharing (66.85%). Half of the respondents (50.90%) preferred short-term intensive courses, and the most commonly preferred session duration was 0.5–1 h for both theoretical (53.58%) and practical (52.33%) training. Weekday afternoons were the most preferred time slot for both theoretical (41.40%) and practical (47.13%) training.

**Table 5:** Educational needs and preferences among willing participants (*n* = 558).

Variable	*N*	%
Theoretical knowledge training requirements (multiple choice)		
Concept of frailty	468	83.87
Epidemiology of frailty	423	75.81
Risk factors for frailty	491	87.99
Adverse outcomes of frailty	469	84.05
Frailty screening	447	80.11
Frailty assessment	489	87.63
Frailty prevention	500	89.61
Frailty intervention	500	89.61
Practical skills training requirements (multiple choice)		
Frailty screening	452	81.00
Frailty assessment	494	88.53
Nutritional intervention for frailty	508	91.04
Exercise intervention for frailty	507	90.86
Health education for frailty	509	91.22
Content to promote learning motivation (multiple choice)		
Nurses’ roles and responsibilities in frailty management	499	89.43
Feasibility and effectiveness of early intervention	516	92.47
Sharing of new guidelines and evidence on frailty	493	88.35
Preferred learning format (single choice)		
Online training courses	135	24.19
Face-to-face training courses	50	8.96
Blended online-offline approach	373	66.85
Preferred learning channels (multiple choice)		
Online information dissemination	427	76.52
Academic conferences and lectures	427	76.52
Hospital-level lectures	362	64.87
Departmental group training	325	58.24
Literature sharing	373	66.85
Consulting relevant experts	288	51.61
Clinical practice accumulation	343	61.47
Sharing of new evidence	262	46.95
Peer communication	261	46.77
Relevant knowledge promotion handbooks	338	60.57
Poster presentations	148	26.52
Preferred training group size (single choice)		
<10 participants	39	6.99
10–20 participants	127	22.76
21–30 participants	72	12.90
31–40 participants	34	6.09
41–50 participants	23	4.12
>50 participants	88	15.77
No preference	175	31.36
Expected training frequency (single choice)		
Short-term intensive courses	284	50.90
2–3 times per week	11	1.97
Once per week	39	6.99
Once every 2 weeks	26	4.66
Once per month	185	33.15
Other	13	2.33
Expected duration for theoretical training sessions (single choice)		
0.5–1 h	299	53.58
1–2 h	186	33.33
>2 h	18	3.23
No preference	54	9.68
Other	1	0.18
Expected duration for practical training sessions (single choice)		
0.5–1 h	292	52.33
1–2 h	187	33.51
>2 h	25	4.48
No preference	53	9.50
Other	1	0.18
Preferred time slots for theoretical training (single choice)		
Weekday mornings	117	20.97
Weekday midday	34	6.09
Weekday afternoons	231	41.40
Weekday evenings	94	16.85
Weekends	73	13.08
Other	9	1.61
Preferred time slots for practical training (single choice)		
Weekday mornings	113	20.25
Weekday midday	36	6.45
Weekday afternoons	263	47.13
Weekday evenings	65	11.65
Weekends	73	13.08
Other	8	1.43

## 4 Discussion

To the best of our knowledge, no multicenter study has comprehensively examined nurses’ KAP regarding frailty together with their educational needs specifically in southwestern China, although single-center or regional studies exist. In this large, multicenter sample of tertiary-hospital nurses, we found moderately high overall KAP (81.16% of the maximum score), but a consistent hierarchy emerged: attitudes (88.53%) exceeded knowledge (81.38%), which in turn exceeded practice (74.40%). After accounting for hospital-level clustering and mutual adjustment, KAP was most consistently associated with two potentially modifiable exposures—recency of frailty education and of frailty-related reading—and, for knowledge, with geriatric nurse specialist certification, whereas fixed demographic attributes were not. Consistent with the exploratory design, associations are interpreted descriptively; the most consistent signal linked more recent education to higher knowledge. Willingness to undertake training was high (91.48%). Because the design is cross-sectional, these factors are best read as modifiable correlates of frailty-care competency rather than causes—a caveat that applies throughout and is not repeated below.

### 4.1 Overall KAP status

As summarized above, favorable attitudes coexisted with the lowest relative score for practice, indicating that adequate understanding and positive attitudes did not consistently translate into clinical action; because the domains use different scales, we interpret this discordance qualitatively rather than as a numerical gap. This pattern is consistent with frailty nursing research across settings (16, 18, 20, 21) and with international reports of under-training—Warnier et al. (15) found that Dutch nurses lacked formal training and did not use screening tools as intended, and Frost et al. (25) reported that only 43% of UK professionals had received frailty training. Compared with the cohorts of Huang et al. (18) and Xuan and Liu (16), our nurses scored somewhat higher across domains, yet practice remained the weakest domain in all of them, suggesting that cognitive competence alone is insufficient to drive implementation.

The recurrence of this pattern points to systemic rather than individual deficits. Organizational barriers recur across studies—inadequate institutional support (52.1%), limited guideline familiarity (17.0%), and low frailty-care training rates (10.5%) in Huang et al. (18) and only 24% of UK facilities with care pathways and 45% with information-sharing systems (25)—suggesting that standardized workflows, adequate resources, and integrated information systems matter at least as much as individual knowledge (15). Interventions should therefore pair targeted education (leveraging nurses’ favorable attitudes) with systemic change: embedding screening into routine workflows with electronic prompts, standardized protocols, adequate staffing, and clear multidisciplinary pathways—consistent with Huang et al.’s (18) finding that organizational support is associated with practice.

### 4.2 Factors associated with nurses’ knowledge, attitudes, and practices

In multivariable models, shorter intervals since the last frailty education and the last reading of frailty-related literature were associated with higher total KAP and knowledge scores, whereas associations with the attitude and practice subscales were weaker or non-significant. Because the design is cross-sectional, these associations cannot establish that more frequent knowledge updates cause higher competence; reverse causation—whereby more competent or more motivated nurses pursue training and reading more often—is equally plausible.

#### 4.2.1 Recency of frailty education

The graded association between recency of frailty education and KAP—with lower scores among nurses trained less recently or not at all (overall *p* < 0.05)—aligns with previous studies. Frost et al. (25) found that increased training supports healthcare professionals in frailty management. Xuan and Liu (16) demonstrated that relevant training experience influences KAP scores among nurses in geriatric-related departments.

Because our cross-sectional data cannot establish mechanism, the following pathways are drawn from the wider literature and are hypothesized rather than demonstrated. Regular training has been proposed to act through several mechanisms: it may consolidate and update knowledge while strengthening beliefs about early identification and intervention and encouraging skill application (26), provides timely access to current guidelines and evidence that broaden perspectives and build confidence in complex cases (27), and strengthens multidisciplinary collaboration so that nurses act more effectively as care coordinators (28).

Based on these findings, healthcare institutions should provide regular, periodic refresher training to help sustain competence; our data cannot identify an optimal training interval. This systematic approach, unlike one-time orientation programs, creates sustained learning platforms that foster an organizational learning culture. Nursing administrators should allocate protected time for education and integrate training participation into performance evaluation systems.

#### 4.2.2 Recency of frailty-related literature reading

Similarly, more recent reading of frailty-related literature was associated with higher KAP, most clearly for the knowledge domain, although—given the exploratory design and the number of comparisons—this should be interpreted with caution. This is nonetheless consistent with Huang et al. (18), who found that guideline familiarity influences frailty-management KAP.

Because the design is cross-sectional, the following interrelated pathways are hypothesized rather than demonstrated. Reading may help establish systematic knowledge and rectify misunderstandings that equate frailty with normal aging or physical disability (19, 29). Such progress consolidates nurses’ recognition of the importance of early frailty screening and intervention, keeps them updated with the latest research evidence and clinical guidelines, and further boosts their confidence in clinical decision-making for complex cases involving older adults (30). These improvements can be transformed into standardized frailty assessment and targeted individualized nursing interventions (31), and also facilitate effective communication and professional collaboration within multidisciplinary medical teams (32).

Therefore, healthcare institutions should integrate frailty literature reading into continuing education programs by establishing resource libraries, organizing journal clubs, and facilitating regular literature sharing sessions to promote sustained reading habits and continuous KAP improvement among nursing staff (28, 33).

#### 4.2.3 Geriatric nurse specialist certification

Geriatric nurse specialist certification was independently associated with higher knowledge but not with attitude or practice. This dissociation implies that current certification effectively transmits cognitive content but does not, by itself, change bedside behavior. This reinforces the view that didactic instruction must be coupled with skills rehearsal, case-based application, and workflow integration to produce behavioral change (34). Specifically, incorporating simulation-based training, practice audits, and case-based reasoning into certification programs may better translate knowledge into practice. The professional-title differences seen in unadjusted analyses attenuated after adjustment: such gradients appear to operate largely through differential access to recent education and literature.

### 4.3 Educational needs and program development strategies

This study found that 91.48% of surveyed nurses expressed willingness to participate in frailty management training, indicating strong learning motivation. This finding is consistent with cross-sectional studies by Xuan and Liu (16) and Frost et al. (25), confirming that high willingness to learn about frailty management represents a prevalent trend in clinical settings. This high willingness aligns with the growing clinical attention to geriatric frailty and lays a foundation for targeted training system development (33).

Regarding training content, most respondents demonstrated high demand for both theoretical knowledge (frailty intervention, prevention, and contributing factors) and practical skills (health education, nutritional management, and exercise intervention). This dual demand reflects the clinical reality that healthcare professionals require not only systematic theoretical foundations but also practical competencies directly applicable to clinical settings (35). As Huang et al. (18) noted, the disconnect between knowledge and practice undermines frailty care competency, underscoring the necessity of integrating both dimensions in training programs. Analysis of learning motivation revealed that the feasibility of early intervention, nursing role responsibilities, and emerging guidelines with evidence-based practice emerged as primary drivers, suggesting that “practical utility” and “evidence-based orientation” are key elements in sustaining engagement (36). Therefore, effective training programs should prioritize balanced integration of theoretical and practical components with emphasis on hands-on skill development (31), utilize case-based teaching to connect theory with clinical practice, incorporate current guidelines to ensure content timeliness, and clarify role delineation among healthcare professionals to establish clear learning objectives and clinical application expectations (37).

Concerning format and logistics, more than two-thirds of respondents favored a blended online–offline model, reflecting a dual demand for flexibility and interactivity: online modules enhance accessibility beyond spatiotemporal constraints, while offline components provide hands-on training and real-time feedback (38). Academic conferences, specialized lectures, and online dissemination were also well-received, underscoring the importance of content authority, timeliness, and format flexibility. For scale and duration, a substantial proportion favored small classes of 10–20 participants (22.76%, the most-chosen specific size, although 31.36% expressed no preference) to allow effective hands-on teaching (39), while about half favored short-term intensive courses (50.90%), and a similar proportion preferred sessions of 0.5–1 h (theoretical 53.58%, practical 52.33%) to fit clinical workload (40). For scheduling, weekday afternoons showed the highest acceptability (theoretical 41.40%, practical 47.13%), with lower acceptance for evenings and weekends, indicating that training should align with clinical schedules and avoid rest periods to minimize participation barriers.

## 5 Limitations

Several limitations, common to cross-sectional survey research, should be borne in mind when interpreting these findings. First, the design supports association rather than causation, and the links with recency of education and reading could partly reflect reverse causation or self-selection. Second, recruitment relied on an online convenience sample coordinated through head nurses (a possible source of managerial influence), so the 95.76% represents a valid-completion rate rather than a true response rate, and nurses more engaged with frailty care—together with those willing to be trained, to whom the educational-preference analysis was necessarily confined—may be over-represented. Third, because participants came from tertiary general hospitals in two southwestern provinces and were nested within 41 sites, generalizability to other settings is limited and residual contextual confounding cannot be fully excluded, although the models were adjusted for within-hospital clustering. Fourth, the self-reported measures are subject to social-desirability and recall bias; and, although the KAP instrument was previously validated, independent validation in other populations remains limited, its knowledge domain combines self-perceived and objectively scored items, and its percentage scores (which have unequal floors across domains) were used for description only, while—consistent with the study’s exploratory aims—associations were not corrected for multiple testing, so category-level contrasts remain hypothesis-generating. The educational-needs items, moreover, formed a preference inventory developed for descriptive purposes that did not undergo formal content-validity quantification or pilot testing. Rather than undermining the findings, these considerations set the agenda for future work: longitudinal or controlled studies using probability sampling, further validated instruments, and objective measures of practice and patient outcomes are needed to confirm the associations identified here.

## 6 Conclusion

Among nurses in tertiary general hospitals in southwestern China, attitudes toward frailty management were positive whereas self-reported practice corresponded to the lowest percentage of its maximum. In this cross-sectional study, more recent frailty education and more recent reading of frailty-related literature were associated with selected KAP outcomes and represent potentially modifiable correlates; no causal or optimal-frequency conclusions can be drawn from these data. Willingness to be trained was high, and respondents expressed clear preferences for blended, practice-oriented learning. These findings can inform the design of continuing-education programs, but longitudinal and controlled studies that incorporate organizational and contextual factors and that use objective measures of practice and patient outcomes are required before firm recommendations about the content, format, or frequency of training can be made.

## Data Availability

The original contributions presented in the study are included in the article/Supplementary material, further inquiries can be directed to the corresponding author.
