# Supplementary material for: Nurses’ knowledge, attitudes, practices, and educational needs regarding frailty: a multicenter cross-sectional survey in southwestern China
Source: Front Public Health. 2026 Sep 17;14:1920309. doi: 10.3389/fpubh.2026.1920309 (PMC13629414; doi:10.3389/fpubh.2026.1920309)
Supplement: Supplementary file 1 [file Supplementary_file_1.docx]

Supplementary Table 1 The Frailty Knowledge, Attitude, and Practice (KAP) Questionnaire for nurses (English translation).

| **No.** | **Item** |
| --- | --- |
| **Domain I — Frailty knowledge, self-perceived items (K1–K6).** *Response options: ① Know (3) · ② Know a little, but not exactly (2) · ③ Do not know (1).* | |
| **K1** | Do you know the concept of frailty? |
| **K2** | Do you know the clinical manifestations of frailty? |
| **K3** | Do you know how frailty is diagnosed? |
| **K4** | Do you know how to assess patients with frailty? |
| **K5** | Do you know the risk factors for frailty? |
| **K6** | Do you know the preventive measures for frailty? |
| **Domain I — Frailty knowledge, objective items (K7–K14).** *Response options: ① Correct (3) · ② Uncertain (2) · ③ Incorrect (1).* | |
| **K7** | Frailty is the most common risk factor for physical functional decline and disability. |
| **K8** | Frailty increases the rate of recurrent illness and unplanned readmission, aggravates patients’ suffering, and reduces their quality of life. |
| **K9** | The internationally recognized assessment methods are the Fried frailty criteria and Rockwood’s Frailty Index (FI). |
| **K10** | The clinical manifestations of frail patients are mainly fatigue and exhaustion; reduced physical activity; multimorbidity; and weight loss (a decrease of >10% in body weight within one year). |
| **K11** | Early identification, intervention, and prevention of frailty progression can improve older adults’ quality of life and provide substantial benefits to the individual, the family, and society. |
| **K12** | The incidence of frailty increases with age. |
| **K13** | Effective management of coexisting chronic diseases (multimorbidity) is one of the measures for the comprehensive management of frailty in older adults. |
| **K14** | Promoting appropriate nutrition and physical exercise at an early stage is an effective intervention for preventing or reducing frailty in older adults. |
| **Domain II — Frailty attitude (A1–A8).** *Response options: ① Strongly agree · ② Agree · ③ Neutral · ④ Disagree · ⑤ Strongly disagree (scored 5–1, strongly agree = 5).* | |
| **A1** | I believe my own knowledge of frailty in older adults needs to meet clinical requirements. |
| **A2** | I believe nurses should continuously monitor the frailty status of older patients. |
| **A3** | I believe nurses should receive formal training on frailty in older adults. |
| **A4** | I believe nurses should take responsibility for the nursing assessment of frailty in older adults. |
| **A5** | I believe early functional exercise is very important for the prevention of and recovery from frailty in older adults. |
| **A6** | I believe healthcare professionals should attach as much importance to preventing frailty in older adults as they do to other conditions (e.g., deep vein thrombosis, DVT). |
| **A7** | I believe that, in clinical practice, patients or their families should be educated about frailty in older adults. |
| **A8** | I believe the frailty status of older patients should be included in clinical shift handovers. |
| **Domain III — Frailty practice (P1–P9).** *Response options: ① Always · ② Often · ③ Sometimes · ④ Rarely · ⑤ Never (scored 5–1, always = 5).* | |
| **P1** | In clinical practice, do you proactively pay attention to patients’ frailty status? |
| **P2** | In clinical practice, do you talk with patients about their limb muscle strength? |
| **P3** | In clinical practice, do you perform frailty assessment for patients? |
| **P4** | Do you promptly report patients’ muscle-strength status to the physicians in your department? |
| **P5** | Do you provide effective early functional-exercise guidance for older patients? |
| **P6** | Do you instruct family members to help patients undertake appropriate activity to relieve physical fatigue and related symptoms? |
| **P7** | Do you promptly evaluate patients after nursing interventions for early mobilization? |
| **P8** | Do you promptly evaluate patients after implementing nutritional interventions? |
| **P9** | Do you accumulate frailty-related knowledge in the course of your work? |

KAP, knowledge, attitude, and practice; FI, Frailty Index; DVT, deep vein thrombosis. The questionnaire was administered in Chinese; the English wording shown here was translated by the authors for presentation and was not itself re-validated.

*Scoring.* The instrument contains 31 items across three domains: knowledge (14 items: 6 self-perceived and 8 objective; domain score 14–42), attitude (8 items; domain score 8–40), and practice (9 items; domain score 9–45). Each item is scored from 1 to the maximum shown in parentheses in the corresponding domain header. The total KAP score ranges from 31 to 127, with higher scores indicating better competency. Item numbering restarts within each domain in the original instrument; the K/A/P prefixes are added here for reference only.

*Source.* This instrument was originally developed and validated in Chinese (reference [16]). It is used in this study for non-commercial academic purposes, strictly following the original version with full citation of the original work.

Supplementary Table 2 Distribution of respondents and knowledge, attitude, and practice scores by hospital (*n* = 610).

| **Hospital** | **n (%)** | **Total KAP score** | **Knowledge** | **Attitude** | **Practice** |
| --- | --- | --- | --- | --- | --- |
| The First Affiliated Hospital of Chongqing Medical University | 260 (42.62%) | 101.46 ± 12.21 | 33.74 ± 3.69 | 34.98 ± 4.56 | 32.74 ± 7.35 |
| Dianjiang People’s Hospital of Chongqing | 62 (10.16%) | 102.85 ± 14.09 | 33.05 ± 4.99 | 35.35 ± 5.25 | 34.45 ± 7.85 |
| Chongqing University Three Gorges Hospital | 38 (6.23%) | 101.34 ± 13.67 | 34.42 ± 4.82 | 36.58 ± 4.08 | 30.34 ± 8.74 |
| Chongqing Changshou District People’s Hospital | 24 (3.93%) | 104.13 ± 11.70 | 33.79 ± 3.46 | 35.67 ± 4.66 | 34.67 ± 7.46 |
| Chongqing Kaizhou District People’s Hospital | 19 (3.11%) | 105.32 ± 14.26 | 34.63 ± 6.34 | 35.26 ± 5.48 | 35.42 ± 5.15 |
| Yilong County General Hospital | 18 (2.95%) | 105.17 ± 11.00 | 34.17 ± 3.71 | 36.11 ± 4.71 | 34.89 ± 6.34 |
| Chongqing Liangping District People’s Hospital | 18 (2.95%) | 97.22 ± 15.34 | 32.50 ± 3.73 | 34.17 ± 5.03 | 30.56 ± 9.95 |
| Chongqing Rongchang District People’s Hospital | 18 (2.95%) | 103.22 ± 12.54 | 34.83 ± 4.95 | 34.33 ± 4.75 | 34.06 ± 6.04 |
| The Seventh People’s Hospital of Chongqing | 14 (2.30%) | 111.14 ± 12.18 | 37.00 ± 6.24 | 38.07 ± 3.38 | 36.07 ± 9.27 |
| Chongqing Dazu District People’s Hospital | 13 (2.13%) | 111.85 ± 9.47 | 36.15 ± 3.18 | 38.00 ± 3.39 | 37.69 ± 5.56 |
| Nanbu County People’s Hospital | 11 (1.80%) | 106.18 ± 9.22 | 35.91 ± 3.56 | 35.18 ± 4.02 | 35.09 ± 5.41 |
| Chongqing University Fuling Hospital | 10 (1.64%) | 110.30 ± 13.64 | 36.50 ± 4.35 | 37.30 ± 3.65 | 36.50 ± 9.57 |
| Nanchong Mental Health Center | 9 (1.48%) | 108.00 ± 12.58 | 37.00 ± 2.96 | 35.67 ± 4.72 | 35.33 ± 7.07 |
| Chongqing Songshan Hospital | 9 (1.48%) | 101.00 ± 9.37 | 31.00 ± 3.20 | 35.56 ± 5.15 | 34.44 ± 6.48 |
| The Seventh People’s Hospital of Chengdu | 8 (1.31%) | 105.88 ± 7.30 | 35.50 ± 2.39 | 36.63 ± 4.37 | 33.75 ± 3.73 |
| Yilong County People’s Hospital | 8 (1.31%) | 104.00 ± 13.78 | 35.25 ± 2.92 | 35.13 ± 5.84 | 33.63 ± 6.25 |
| Chongqing University Jiangjin Hospital | 8 (1.31%) | 108.75 ± 11.07 | 35.38 ± 3.20 | 35.75 ± 4.62 | 37.63 ± 4.60 |
| Affiliated Yongchuan Hospital of Chongqing Medical University | 8 (1.31%) | 102.13 ± 6.40 | 35.88 ± 4.02 | 35.88 ± 2.75 | 30.38 ± 4.69 |
| Guangyuan Central Hospital | 7 (1.15%) | 110.29 ± 10.58 | 35.00 ± 3.96 | 37.00 ± 3.87 | 38.29 ± 5.28 |
| Chongqing Yongchuan District People’s Hospital | 5 (0.82%) | 98.00 ± 16.40 | 32.00 ± 2.92 | 34.40 ± 7.13 | 31.60 ± 8.20 |
| Chongqing Hechuan District People’s Hospital | 4 (0.66%) | 100.25 ± 6.85 | 36.00 ± 0.00 | 32.75 ± 2.87 | 31.50 ± 4.65 |
| Guang’an Hospital of West China Hospital, Sichuan University | 4 (0.66%) | 101.25 ± 5.12 | 32.75 ± 1.90 | 34.00 ± 3.16 | 34.50 ± 1.29 |
| Nanchong Central Hospital | 3 (0.49%) | 119.67 ± 11.85 | 38.67 ± 4.93 | 38.67 ± 2.31 | 42.33 ± 4.62 |
| Panzhihua Central Hospital | 3 (0.49%) | 97.67 ± 12.01 | 35.33 ± 1.53 | 34.00 ± 2.65 | 28.33 ± 8.62 |
| Zhongxian People’s Hospital | 3 (0.49%) | 99.33 ± 11.93 | 33.33 ± 3.06 | 40.00 ± 0.00 | 26.00 ± 9.54 |
| Chongqing General Hospital | 3 (0.49%) | 107.67 ± 15.57 | 37.33 ± 4.16 | 33.67 ± 4.73 | 36.67 ± 8.50 |
| The Second Affiliated Hospital of Chongqing Medical University | 3 (0.49%) | 114.00 ± 12.12 | 38.33 ± 3.21 | 37.00 ± 5.20 | 38.67 ± 5.51 |
| Wusheng County People’s Hospital | 2 (0.33%) | 89.50 ± 16.26 | 29.50 ± 2.12 | 33.00 ± 1.41 | 27.00 ± 12.73 |
| Yuechi County People’s Hospital | 2 (0.33%) | 105.50 ± 10.61 | 35.50 ± 2.12 | 34.50 ± 7.78 | 35.50 ± 0.71 |
| The Thirteenth People’s Hospital of Chongqing | 2 (0.33%) | 110.00 ± 0.00 | 40.00 ± 2.83 | 34.50 ± 3.54 | 35.50 ± 0.71 |
| Chongqing Yunyang County People’s Hospital | 2 (0.33%) | 106.50 ± 0.71 | 37.50 ± 2.12 | 38.00 ± 2.83 | 31.00 ± 0.00 |
| Jiangsu Province People’s Hospital Chongqing Hospital | 2 (0.33%) | 105.00 ± 11.31 | 33.00 ± 7.07 | 34.50 ± 3.54 | 37.50 ± 0.71 |
| The Ninth People’s Hospital of Chongqing | 2 (0.33%) | 117.50 ± 0.71 | 39.00 ± 2.83 | 36.50 ± 0.71 | 42.00 ± 1.41 |
| Bazhong Central Hospital | 1 (0.16%) | 95.00 | 31.00 | 32.00 | 32.00 |
| The First People’s Hospital of Guangyuan | 1 (0.16%) | 97.00 | 28.00 | 35.00 | 34.00 |
| Linshui County People’s Hospital | 1 (0.16%) | 104.00 | 36.00 | 32.00 | 36.00 |
| Xinqiao Hospital of Army Medical University | 1 (0.16%) | 112.00 | 36.00 | 40.00 | 36.00 |
| Army Medical Center of PLA | 1 (0.16%) | 78.00 | 28.00 | 23.00 | 27.00 |
| Chongqing Qijiang District People’s Hospital | 1 (0.16%) | 127.00 | 42.00 | 40.00 | 45.00 |
| West China Hospital, Sichuan University | 1 (0.16%) | 75.00 | 35.00 | 31.00 | 9.00 |
| Chonggang General Hospital | 1 (0.16%) | 99.00 | 34.00 | 38.00 | 27.00 |

Values are mean ± SD unless otherwise indicated. KAP, knowledge, attitude, and practice. For hospitals with a single respondent (*n* = 1), only the observed value is shown because the SD is not defined. Percentages are calculated with *n* = 610 as the denominator and may not sum to 100 owing to rounding. Hospital names reported by respondents were checked and standardised: entries referring to the same institution under different names were merged, whereas institutions operating as separate campuses or independent legal entities were retained as distinct sites. The resulting 41 sites were used as the clustering unit in the mixed-effects models.

Supplementary Table 3 Distribution of respondents and knowledge, attitude, and practice scores by clinical department (original classification; *n* = 610).

| **Department** | **n (%)** | **Total KAP score** | **Knowledge** | **Attitude** | **Practice** |
| --- | --- | --- | --- | --- | --- |
| Nephrology | 74 (12.13%) | 105.04 ± 12.27 | 34.11 ± 3.77 | 36.39 ± 4.05 | 34.54 ± 8.01 |
| Other/Not Specified | 71 (11.64%) | 101.20 ± 14.91 | 34.24 ± 4.83 | 35.90 ± 4.67 | 31.06 ± 9.53 |
| General Practice | 67 (10.98%) | 106.67 ± 11.12 | 35.10 ± 4.07 | 35.90 ± 3.92 | 35.67 ± 6.27 |
| Oncology | 59 (9.67%) | 102.88 ± 13.78 | 34.15 ± 4.10 | 35.20 ± 4.57 | 33.53 ± 7.98 |
| Neurology | 43 (7.05%) | 102.70 ± 11.63 | 33.12 ± 3.42 | 33.95 ± 4.98 | 35.63 ± 5.72 |
| Hematology | 37 (6.07%) | 99.57 ± 11.65 | 33.70 ± 3.08 | 35.43 ± 4.41 | 30.43 ± 8.78 |
| Cardiology | 32 (5.25%) | 101.06 ± 10.84 | 34.66 ± 4.08 | 33.91 ± 4.35 | 32.50 ± 5.84 |
| Respiratory Medicine | 25 (4.10%) | 106.92 ± 12.66 | 35.00 ± 3.57 | 36.00 ± 4.47 | 35.92 ± 7.50 |
| Orthopedics | 20 (3.28%) | 106.70 ± 12.69 | 35.50 ± 4.41 | 35.45 ± 4.05 | 35.75 ± 6.36 |
| Neurosurgery | 20 (3.28%) | 104.75 ± 14.28 | 33.80 ± 5.85 | 35.20 ± 5.60 | 35.75 ± 5.41 |
| Gynecology | 16 (2.62%) | 100.13 ± 14.18 | 33.06 ± 2.77 | 34.69 ± 5.42 | 32.38 ± 7.44 |
| Infectious Diseases | 16 (2.62%) | 97.25 ± 11.84 | 34.19 ± 3.54 | 31.63 ± 4.83 | 31.44 ± 6.35 |
| Geriatrics | 14 (2.30%) | 105.50 ± 11.41 | 36.79 ± 4.19 | 36.43 ± 3.44 | 32.29 ± 7.32 |
| Otorhinolaryngology | 12 (1.97%) | 100.17 ± 8.56 | 31.58 ± 3.92 | 36.00 ± 4.41 | 32.58 ± 4.66 |
| Gastroenterology | 12 (1.97%) | 99.58 ± 10.60 | 33.75 ± 3.41 | 34.58 ± 5.76 | 31.25 ± 5.41 |
| Endocrinology | 11 (1.80%) | 102.00 ± 13.74 | 33.27 ± 7.35 | 36.73 ± 4.38 | 32.00 ± 7.36 |
| Critical Care Medicine | 11 (1.80%) | 100.64 ± 12.53 | 33.64 ± 4.52 | 34.27 ± 5.04 | 32.73 ± 5.14 |
| Nursing Administration | 8 (1.31%) | 101.13 ± 15.18 | 36.13 ± 4.76 | 34.63 ± 5.34 | 30.38 ± 10.10 |
| Emergency Medicine | 8 (1.31%) | 102.75 ± 12.75 | 34.88 ± 1.73 | 35.63 ± 5.37 | 32.25 ± 7.05 |
| Obstetrics | 7 (1.15%) | 101.00 ± 13.78 | 31.29 ± 6.10 | 36.57 ± 4.72 | 33.14 ± 8.57 |
| Gastrointestinal Surgery | 7 (1.15%) | 105.29 ± 11.31 | 35.29 ± 4.54 | 36.86 ± 4.41 | 33.14 ± 4.53 |
| Urology | 6 (0.98%) | 98.50 ± 12.88 | 32.00 ± 3.03 | 32.17 ± 5.53 | 34.33 ± 6.38 |
| Integrated Traditional Chinese and Western Medicine | 6 (0.98%) | 107.83 ± 17.55 | 35.50 ± 5.21 | 35.50 ± 6.38 | 36.83 ± 7.47 |
| Health Management Center | 4 (0.66%) | 93.00 ± 10.03 | 28.00 ± 4.32 | 33.50 ± 8.19 | 31.50 ± 5.20 |
| Ophthalmology | 4 (0.66%) | 112.25 ± 11.30 | 34.75 ± 2.99 | 39.00 ± 1.15 | 38.50 ± 7.55 |
| Rheumatology and Immunology | 3 (0.49%) | 109.67 ± 10.69 | 36.00 ± 5.29 | 36.67 ± 1.53 | 37.00 ± 4.36 |
| Oral and Maxillofacial Surgery | 3 (0.49%) | 97.33 ± 12.86 | 36.00 ± 2.65 | 35.00 ± 5.57 | 26.33 ± 5.86 |
| Pain Medicine | 3 (0.49%) | 112.00 ± 5.57 | 36.67 ± 1.53 | 39.67 ± 0.58 | 35.67 ± 4.51 |
| Cardiothoracic Surgery | 3 (0.49%) | 100.00 ± 9.85 | 34.67 ± 2.31 | 38.67 ± 1.53 | 26.67 ± 8.50 |
| Hepatobiliary Surgery | 2 (0.33%) | 99.00 ± 16.97 | 28.00 ± 4.24 | 36.00 ± 4.24 | 35.00 ± 8.49 |
| Psychiatry | 2 (0.33%) | 107.00 ± 1.41 | 35.50 ± 0.71 | 40.00 ± 0.00 | 31.50 ± 2.12 |
| Vascular Surgery | 2 (0.33%) | 96.00 ± 2.83 | 33.00 ± 2.83 | 31.00 ± 1.41 | 32.00 ± 4.24 |
| Rehabilitation Medicine | 1 (0.16%) | 106.00 | 31.00 | 39.00 | 36.00 |
| Breast and Thyroid Surgery | 1 (0.16%) | 101.00 | 31.00 | 34.00 | 36.00 |

Values are mean ± SD unless otherwise indicated. KAP, knowledge, attitude, and practice. “Other” denotes small or unclassified departments. For departments with a single respondent (*n* = 1), only the observed value is shown because the SD is not defined. Percentages use n = 610 as the denominator and may not sum to 100 owing to rounding. For the regression analyses, these departments were collapsed into broader categories (see the main-text tables).
